# Is love an abstract concept? A view of concepts from an interaction-based perspective

**DOI:** 10.1098/rstb.2021.0356

**Published:** 2023-02-13

**Authors:** Joanna Rączaszek-Leonardi, Julian Zubek

**Affiliations:** Human Interactivity and Language Lab, Faculty of Psychology, University of Warsaw, Warsaw, Mazovian 00-183, Poland

**Keywords:** concepts, embodied cognition, ecological psychology, social interaction

## Abstract

Research concerning concepts in the cognitive sciences has been dominated by the information-processing approach, which has resulted in a certain narrowing of the range of questions and methods of investigation. Recent trends have sought to broaden the scope of such research, but they have not yet been integrated within a theoretical framework that would allow us to reconcile new perspectives with the insights already obtained. In this paper, we focus on the processes involved in early concept acquisition and demonstrate that certain aspects of these processes remain largely understudied. These aspects include the primacy of movement and coordination with others within a structured social environment as well as the importance of first-person experiences pertaining to perception and action. We argue that alternative approaches to cognition, such as ecological psychology, enactivism and interactivism, are helpful for foregrounding these understudied areas. These approaches can complement the extant research concerning concepts to help us obtain a more comprehensive view of knowledge structures, thus providing us with a new perspective on recurring problems, suggesting novel questions and enriching our methodological toolbox.

This article is part of the theme issue ‘Concepts in interaction: social engagement and inner experiences’.

## 1. Introduction

‘The baby, assailed by eyes, ears, nose, skin and entrails at once, feels it all as one great blooming, buzzing confusion’ ([1], p. 488). This oft-quoted phrase is understood by many as a description of a point of departure for the process of human learning. The subsequent steps of this process involve learning to distinguish features within this confusion and to organize them into mental structures, which allow us to classify the world into objects and events, and accrue knowledge about them in subsequent experiences.

For some reason, however, most theories of the manner in which such conceptual structures emerge do not take into account the whole experience that is available to an infant. Research concerning concepts in cognitive psychology and cognitive science has been dominated by the analysis of visual experience and subsequent classification based on feature detection, a tendency that has perhaps been reinforced by advances in computer vision, where deep neural networks have become very successful at various object recognition tasks. This tendency remains evident in recent experimental works pertaining to concept learning (e.g. [2–4]), in which static visual stimuli representing objects with well-defined features have continued to prevail.

However, even a cursory look at most moments in the day of a developing infant reveals that her experience is incomparably richer. An infant is active all the time, and her activity causes changes in the environment that serve as the basis of perception. She is in contact with the world through all her senses *at once*, and everything that she perceives remains related to the constant presence of the feelings generated by her own body, including her sense of movement. Importantly, all actions and perceptions occur in a predominantly social world, which is responsive to and engages the infant by means of intricate and culturally shaped enactments. For example, Nomikou *et al*. [5] discuss the way in which an infant's ability to time her actions in the context of social routines, such as the game of peekaboo, provides experiences to facilitate an understanding of such routines. External scaffolding, by enabling participation, guides infants' attention and perception.

A significant body of work has already shown evidence of the importance of relational features [6], goal- and action-dependent situational properties [7–9] and interoception [10,11] for shaping concepts. However, adopting a fundamentally relational, processual, first-person dependent and social approach calls for a deeper reorganization of the field. In this paper, we argue that approaches to cognition that take action and social coaction in the world as a starting point are well suited to develop an account of the richness of experience that underlies concepts.

Such an account has the potential to provide novel perspectives on problems that are recurrent in research concerning concepts. For example, questioning the primacy and dominance of visual experience leads to a reconsideration of the meaning of the concrete–abstract dimension. Similarly, the distinction between concepts concerning objects (entity categories) and concepts concerning relations (relational categories) becomes more nuanced, and the primacy of objects is put into question. The main types and levels of categorization can be more firmly based in individual and social learning trajectories. Noting the continuous presence and significance of first-person experience offers the possibility of a fuller integration of structures of knowledge with affective and normative engagements within the social world. Crucially, novel methods can be employed to study concepts by recognizing their continuous engagement in and dependence on actions and interactions.

## 2. Concepts in action-first approaches

The most prominent research programs that have recognized the primacy of action for cognition are ecological psychology, enactivism and interactivism. In this paper, we relegate the (sometimes profound^1^) differences among these programs to the background and refer to them collectively as ‘action-first’ or ‘interaction-based’ approaches. In these approaches, embodiment is not viewed as originating in mental representations nor simulations of the body; rather, all knowledge structures are derived from embodied interaction with the world [13–17]. Action-dependent, goal-oriented, affordance-based knowledge organization and grounding in first-person experience are among the basic tenets of action-first approaches (albeit to different degrees). Their philosophical foundations in radical empiricism (most strongly acknowledged in the case of ecological psychology [18]) help clarify the relations among perceiving, acting and conceptualizing.

Perception is not demarcated as a precognitive substrate for cognition. It is not a supply of input that must be ‘ordered’ by its incorporation into concepts understood as knowledge structures. Both perceiving and conceptualizing are ways of knowing (see also [19]). Perceiving and conceiving are processes that are based on selection and differentiation among potential relationships between the agent and the environment, and they lead to the discovery of the latent structure of agent–world relations.

Percepts and concepts interpenetrate … Neither, taken alone, knows reality in its completeness. We need them both, as we need both our legs to walk with ([20], 53).

Knowing is thus not the addition of well-defined, finite facts or contents to an individual store in order to use them later to enrich sensations. Knowledge becomes manifest in changes in perception. The organism attunes itself to making new distinctions, selecting them from latent possibilities (i.e. the distinctions that are possible for a given organism to make at all). A cognitive system learns to perceive the world in a way that is useful for its present and future actions [21]. Cognition is an anticipatory, forward-looking process rather than a process based on accruing and deploying knowledge of the past, which is stored in the form of a finite internal model of certain content [16]. On such a view, perception, including complex relations and temporal patterns, is direct, guided by multiple goals, projects and values serving as constraints at any given moment [22,23]. The contextual flexibility is not the result of an adaptation of otherwise stable internal structures to external conditions but rather is due to the fact that the environment is an inherent and constitutive part of perceptual and conceptual knowledge.

This action-oriented framework does not preclude the possibility that humans can engage in highly abstract conceptual reasoning, which requires the extraction of contextually stable relations (as in the case of formal systems, taxonomies or general definitions). Simultaneously, we are able to use flexible, context-dependent concepts and to form novel concepts at need. We use concepts to drive movement-based decisions, and our knowledge is drawn from our bodily interactions with the world and never truly decoupled from that world. Our movements are gentler when we hold a brittle or precious cup than when we hold a common plastic cup. Even engaging in abstract deductions when proving a mathematical theorem elicits certain feelings, such as frustration or elation, which are experienced via the body.

Such a framework requires us to revise the assumptions, which are tacitly endorsed by many works concerning concepts, that ‘input’ can be reduced to certain features as atoms of meaning and that these features originate in individual cognition of the external world. In the following, we develop the consequences of accepting the main tenets of action-based frameworks for the theory of concepts: (i) recognizing the fundamentally relational nature of concepts, which is dictated by the pragmatic nature of cognition; (ii) emphasizing the importance of first-person experience in concept emergence and use; and (iii) comprehending the vital role of social coordination.

### (a) The fundamentally relational nature of concepts: it's relations ‘all the way down’

According to the approach of radical empiricism, perception takes the form of a differentiation of the field of experience along the ‘lines of order intrinsic to the field’^2^ ([18], p. 38). Perception does not start with unorganized features that must be detected as atoms and assembled to form coherent, sensible percepts. The key idea embraced by ecological psychology and largely shared by other interaction-based approaches is that relations are perceived directly, as they are part of the anticipatory organization of the cognitive system that were stabilized while acting in a given environment.

As Heft [18] rightly notes, James's ‘blooming, buzzing confusion’ is rarely quoted in its significant context, which precludes an atomistic reading. For example, in *The principles of psychology* ([1], p. 488), the phrase is used as follows: ‘The baby, assailed by eyes, ears, nose, skin and entrails at once, feels it all as *one* great blooming, buzzing confusion; and to the very end of life, our location of all things in one space is due to the fact that the original extents or bignesses of all the sensations which *came to our notice at once, coalesced together into one and the same space*. There is no other reason than this why ‘the hand I touch and see coincides spatially with the hand I immediately feel.’ (Montgomery, Mind, p. 527)’ (emphasis added). James notes the fact that this experience is one, thus highlighting the feeling of its being here and now and its potential possession of an order. Moreover, experience is normatively adaptive in its unity, as specified in another use of the phrase: ‘If my reader can (…) lapse back into his immediate sensible life at this very moment, he will find it to be what some one has called a big blooming buzzing confusion, as *free from contradiction in its much at-onceness as it is all alive and evidently there*’ ([20], p. 50, emphasis added; see also [18], p. 39, and [24]). Order is thus not imposed by the individual mind but rather discovered or individuated within the existing relation between the knower and the known.

There are at least two crucial ways in which relationality is associated with the understanding of perception and cognition developed by radical empiricism. First, what is directly perceived are ‘affordances’—relational properties of an organism in its environment, which can guide possible actions ([13]; see [18], pp. 123–135). Instead of perceiving the objective shape and size of a chair, an agent notices that the shape and size are just right to offer the possibility of sitting. The basic relation of the pertinence of the world to an agent cannot and does not have to be constructed based on certain ‘objective’ features. Second, for an organism whose perception is tuned to dynamic interactions with the world, perceived affordances are themselves complex structures. When trying to catch a moving object, e.g. a frog hopping between leaves, the rate of change in the occlusions of one object by the other is crucial to calibrate an action. Thus, what is perceived in the context of the relation to the self is relationally structured in its own right.

The relational nature of concepts features prominently in some research in cognitive psychology concerning concepts. As Dedre Gentner (e.g. [6,25]) writes in one of her seminal papers: ‘*Fish gotta swim and birds gotta fly. Robins eat worms, dogs chase cats, roses love sunshine. These kinds of relations are as much a part of our understanding of the world as are the direct properties of the entities themselves*' ([25], p. 245). Gentner distinguishes entity categories from relational categories in a manner similar to the traditional division and recognizes complex structures in the external world, which corresponds roughly to the second sense of relationality. Action-first approaches additionally emphasize the primary relationality of perception and the continuity of perception and cognition, both of which serve action. Since relations are perceived directly, the difference between the relational properties and perceptual properties of ‘simple’ objects and features ceases to be obvious. This claim may be harder to accept against the backdrop of theories of conceptual representations in cognitive science that are founded on the objective-feature-based nature of ‘data’ or ‘stimuli’.

Despite these theoretical disparities, Gentner's descriptions of the processes that can lead to the development and use of relational categories can be interpreted in terms of radical empiricist notions, such as direct perception of relations, action-orientation, prospective/anticipatory directedness and selectivity. Some of Gentner's own examples come very close to the interactivist view of concepts that we advocate here. Among the dimensions along which relational concepts are developed, she includes spatio-temporal juxtaposition, such as in the case of actions in time; a child's behaviour of repetitively constructing a block tower and knocking it down ‘may reflect their delight at comparing and learning from small variations in the action’ ([25], p. 259). It can easily be imagined that some of the novel relations that are noticed and used to categorize events might involve complex, dynamical variables (for example, the dynamics of ‘chase’, similar in the case of dog chase and shark chase, which helps understand the concept [25]; for similar dynamics-based understanding of complex concepts see also [26]).

### (b) The importance of first-person experience: it's experience ‘all the way out’

The fundamentally relational nature of cognition, which is evident at the point of connection between agents and their environment, simultaneously draws researchers' attention to the world and, crucially, to first-person experiences. Affordances are always relations between the world and the acting organism, which manifest the presence of both and broaden the spectrum of ‘perceivables’ to include the properties of self-movement, felt movements and affectivity.

Ecological psychology highlights the importance of one's own perceivable movement and aspects of proprioception, while other researchers, such as Sheets-Johnstone [27], detail the richness of experience connected to movement. The self-felt movement, imbued with affectivity, constitutes the ‘relata’ to experiences in other modalities, thus contributing to Jamesian oneness or all-in-oneness. This relatedness of perception to self-motion and inner experience of moving is missing from most theories of concepts in the field of cognitive science, the focus being on the outside world. However, as perceived by a living agent, the inner world is indisputably there. Both the inner world and the outer world are continuously present for a cognitive agent, ready for use in navigating the world and constructing explanations, which reduces the need to engage in inferencing and internal ‘processing’.

The contention that there are no ‘sensations’ from outside that are not simultaneously felt experiences of the body should deeply affect any theory of concepts. Noting that inner experience—whether the lived, felt experience of motion, emotion, image or thought—is an integral part of relations that are relevant to concepts makes even most abstract concepts in contact with the online activity and experience. On this view, it is difficult to think about even the most abstract concepts as purely formal and disembodied. The contact with perception and experience here and now offers the ultimate verification of ‘validity and meaning’, truth ([18], p. 43–44), or the Jamesian road back to perception, which is a necessity for knowledge to become usable and pertinent to our actions in the world.

Importantly, the continuous contact with relational experiencing guarantees the relevance of our knowledge both to the situation at hand and to the individual's history and the hierarchy of values. Concepts superimpose relations on percepts, but percepts supplement concepts. As evident from some of the works within the action-first paradigms, this offers a viable solution to the frame problems that have pervaded most theories of conceptual knowledge representation (see e.g. [28]). In simple terms, conceptual knowledge does not need to be updated explicitly in light of new experiences, since experience itself is the substrate of concepts. Acquiring knowledge means establishing novel working relations that will enable selection and organizing experience along novel dimensions and not that new, explicit content is added. Other means of organization do not vanish, but are latently present ‘so that we may know what is in the wind for us and get ready to react in time’ ([29] p. 31). In such a metastable organization, temporary orders emerge via engagements in tasks and social structures.

Recently, some aspects of first-person experience have been the target of research concerning concepts. According to this research, interoception, which is presented as the ‘sensorimotor grounding’ of the content of mental representation, is a rich source of features that are important for concepts. For example, interoception is claimed to ‘dominate emotion concepts' and to enrich the abstract–concrete dimension [10]. Borghi and colleagues' work concerning the engagement of various effectors in the evocation of different concepts also highlights the diversity of first-person experiences engaged in conceptualization [11,30]. The stronger reliance of some concepts on language engages experiences that are connected to language (including the motor experiences associated with producing speech), and this process may be responsible for the ease with which novel relations are noticed or formed.

Acknowledging the omnipresence of the first-person experience and the primary relationality for all kinds of concepts may provide a good framework to link these findings to the action-first paradigms. It will motivate researchers to seek other types of first-person experiences that may be important for particular kinds of concepts and investigate their dependence on social interactions within cultural routines. It may give new meaning, or new ordering factors to the dimensions, which usually serve to characterize concepts and which seem to differ vastly with respect the qualities of experience and individual trajectories of acquisition and use that they probe (e.g. imaginability, age of acquisition).

### (c) The human environment is a social–interactive environment: it's social ‘all the way through’

The third change in our habitual thinking about concepts pertains to the nature of the environment of cognitive agents and thus the nature of their first experiences. At least in the domain of cognitive psychology and cognitive science, the perspective typically adopted seems to be that of a scientist, which can be generalized to the universal Umwelt of an adult human being. The environment is populated by identifiable objects and events, which are distinguished by their features (e.g. their shapes, colours or textures) and which exhibit permanence and other properties that are congruent with the laws of physics.

In most action-first approaches, the characterization of the environment^3^ is different. Since the environment is accessible to us via our engagements with it, its dynamical and social nature is highlighted; the environment can be characterized in terms of processes, changes and stabilities rather than in terms of sets of static objects and their features [16]. Focusing on development makes it even more evident just how few physical objects are relevant to action at this stage. The infant's world is instead weaved around her by the presence and actions of other people. The first things in their surroundings on which infants are able to exert influence are not physical objects and their movements but rather social events, which feature a less regular but still predictable spatial and temporal structure and are strongly dependent on infants' own behaviours.

This characteristic context of human activity in the world is obvious for researchers in the domain of cognitive development but still seems to be awaiting its full impact on the theory of the emergence and use of conceptual structures. The works of Wygotsky (e.g. [32]), Stern (e.g. [33]), Trevarthen (e.g. [34]) and Reddy (e.g. [35,36]) make it obvious that the first ‘categorization tasks’ in which a newborn child engages pertain to the relations between the actions of others and the child's felt, bodily experiences of being with another person. The skills first acquired by the child concern her relationships and engagement with others and preserve certain values, namely, those that are crucial for surviving and those that have been culturally selected and stabilized, rather than object categorization, manipulation and the acquisition of encyclopaedic knowledge about the world.

The early environment is structured by other people, i.e. by their actions with specific sequences and timings, which are adjusted to accommodate the actions and rhythms of the infant [36,37]. Participation in early co-action becomes a substrate of conceptual knowledge on a par with perhaps more abstract relations according to which such experiences might subsequently be ordered. Early in development they are arguably even more important than, e.g. information regarding the visual properties of objects, but importantly, this participatory aspect never vanishes. To investigate this highly structured environment in greater detail, it is thus necessary to turn to the microanalyses of such engagements, without assuming that objects, events and relations are obviously and objectively there and instead observing their emergence from repetitive enactments in the context of social routines, i.e. in relation to first-person experiences.

In summary, we propose an understanding of concepts based on the three tenets discussed above as a way of developing a more comprehensive framework. Concepts are created as possibilities for selection from the experiences of an agent in a social environment. Percepts and concepts are located on a continuum rather than on the opposite sides of an imaginary wall. Both percepts and concepts are skills of differentiation based on histories: evolutionary, developmental and interactive. The factor that varies between them is perhaps the degree to which those differentiations are based on individual, bodily, experiential histories or histories of social interaction that are mediated by routines and linguistic and material structures that evolve in cultures and populations. This may offer a fresh perspective on recurring problems in research concerning concepts.

## 3. A view based on the reorganized framework

In order to show the potential of integrating the tenets of the action-first framework with extant research on concepts we have chosen the problem of concrete–abstract division, including the status and concreteness of objects, and the problem of the types and hierarchies of conceptual structures, which might be viewed as the results of different trajectories for selecting relations.

### (a) The concrete–abstract dimension

To understand the process of abstraction, i.e. the path running from the concrete to the abstract, in light of the three claims discussed above, we must reflect on the question of which relations can be considered ‘concrete’ in the first place. Previous views (but see exceptions, e.g. in [9]) have understood as concrete the concepts that correspond to visually identified objects that are describable in terms of sets of features. Being open to the relational nature of every act of perception and highlighting the first-person and social experiences puts such a view into question.

To determine experiences on which abstract relations may be imposed, we shall search for relational experiences that might appear early in development, be closely linked to bodily, affective feelings, and involve social engagements. Infants, who are as yet unable to move around effectively, nevertheless actively explore the possibilities of their own body and learn how to coordinate their movements within their immediate environment, such as turning their heads to look around or rolling from their backs to their stomachs. This embodied experience of self, self-movement and one's own needs accompanies any other experience resulting from contact with other persons and objects. To satisfy their needs, infants rely on relations with their caretakers, which they learn to navigate and control (e.g. [38]).

Early concepts supporting such interactions are thus affectively laden and connected to interoception and the infant's momentary needs. Rather than pertaining to objects within an inanimate world, these concepts are formed on the basis of repeatable patterns of dynamical experiences, such as ‘the particular dynamics of rolling’, ‘a sensation of being enveloped in a warm blanket’, ‘laughing together with a mother’ or ‘making noise with a rattle’. They are concepts in the sense that they are experienced repeatedly and so lead to the formation of relations, which allow for perception to become anticipatory with respect to subsequent interactions (see [39] for an example of a model of dynamical bases of the concept of ‘agency’). Through repeated engagements, the immediate experience is gradually enriched by such relations; for instance, the feeling of hunger becomes embedded in the infant's experiences and control of social relations to form a social concept of hunger, which in turn affects his subsequent experiences.

Such social experience will also guide the use of objects [40], both when prompted by the individual history of objects, such as when we give our baby blanket to a person who is suffering, but also in the context of the everyday use of things. From this perspective, the act of wrapping a baby or a kitten in a soft and warm piece of cloth seems to be a feat that is much less ‘representationally hungry’, and much more naturally normative, being grounded in first-person experiences [41]. Knowledge about such an act relies on different relations from, e.g. knowledge how to construct an airplane, but we are able to engage in both kinds of skills. The differences in the kinds of relations employed result from different trajectories of discovering and selecting relevant aspects of experience infused with social relations, and not from the use of different cognitive modules or different representational forms.

This view of the issue of concreteness and abstractness may help us understand the surprising results that have been reported by recent research concerning concepts. Concepts are assessed on multiple dimensions and clustered to reveal differences and occasionally inconsistencies with the assumed classifications. For example, emotional concepts cluster more closely to concrete concepts than expected [42] and are rated higher with respect to the ability to be experienced or ‘embodied’ [43,44]. Differences are usually explained in accordance with the ‘multiple representations’ view, which tries to account for the variability of results by reference to the information present in those representations. Barsalou, Dutriaux & Scheepers [9] suggested that in a situated context, emotion concepts integrate internal mental states with emotion-eliciting stimuli, which justifies their position in between abstract and concrete. However, according to the processual view presented here, this multiplicity can also be accounted for as the result of differences in the cognitive trajectories of experience with various relations within social interactions.

Broadening perception to encompass first-person, felt experiences would explain this closeness between emotional concepts and the concrete. As much as we agree that concepts such as pain, pleasure and love are, in part, socially constructed, understanding them as abstract concepts and making painstakingly efforts to ground them in experience seems awkward. Their immediate presence in engagements with the world makes such concepts more concrete than even the simplest artefacts. Embedding these structures within novel relations and the increasing coherence of conceptual webs of such relations (which are supported by interactions with others, including linguistic interactions) may make this experiential (emotional, situational, episodic) grounding less apparent, but it never vanishes. As William James noted, such coherence may lead to an ‘everlasting slip, slip, slip, of direct acquaintance into knowledge-about, until at last nothing is left about which the knowledge can be supposed to obtain’ ([45], p. 19), the progressive alignment of new relational situations across contexts results in the various degrees of abstraction and ‘gentrification of knowledge’ [46]. On the present view, what is concrete is closely linked with all experience, including internal experience, and abstraction consists in adding dimensions of selection for the purpose of reorganizing this experience rather than substituting the extant relations with novel ones (including relations among linguistic symbols) entirely.

This view offers hope that the Jamesian slipping of ‘direct acquaintance’ into ‘knowledge-about’ and ‘vicious intellectualism’ ([47], p. 657) or Gentner's ‘gentrification’ of knowledge will never be complete. Among the available moves along the latent structure, it remains possible to unearth connections to immediate experience, to test our concepts against it, to allow the intellect ‘to reinsert itself with its conclusions into some particular point of the immediate stream of life’ ([29], p. 31) and to take ‘reality bodily and integrally up into philosophy in exactly the perceptual shape in which it comes’ ([20], p. 95).

### (b) From relations to objects and back

Our perspective on what is ‘concrete’ suggests that textbook examples of concrete concepts such as ‘ball’, ‘rattle’, ‘chair’ or ‘bottle’ might not fit naturally in this category. By contrast, abstraction might be necessary to generate a concept of a stable object, which must begin with dynamical engagements, and perception of affordances, to which the structures of knowledge pertain. Objective features and objects are abstractions that have become shareable across instances and with others. Starting the analysis of cognitive processes by taking these abstractions as ‘concrete objects’, as is common in the cognitive sciences, actually takes for granted and views as basic what must instead be stabilized in the flow of experience with effort, which is often accomplished through routinized actions, coactions and the structures of language.

Such stabilization undoubtedly has its foundation in our biological preparedness to perceive the physical environment. It is important, however, to highlight the fact that such preparedness takes the form of morphological constraints on processes rather than specific contents: a great deal is left flexible to enable functional interaction with the physical and social world. Thus even if our perceptual systems are prepared to deal with the world of movable objects, the functioning of our perception is also contingent on individual experiences within a particular environment [48]. Early experiences of felt movement and touch in relation to visual experiences can be viewed as sources of relations, which contribute to objects' wholeness and permanence.

Rather than relying on knowledge that is represented in the mind in the form of ‘object files’, which enrich basic perceptions, the process of acquiring knowledge about the world consists of broadening the possibilities of selection. This is enabled by an enrichment of the system of relations via experience of active life in the world and through a growing coherence of a system of relations in a developmental progression [49]. On this view, object permanence, which is often viewed as an indisputable achievement of cognitive maturation, is itself implicated as experience-dependent and even as malleable and prone to disorders, as some authors indicate [50,51].

In recent works in developmental psychology, the stable perception of objects is understood as emerging from multimodal interactions involving objects within social engagements [52,53]. This emergence—as is claimed here—is as much a conceptual process as a perceptual process. Social interactions with objects individuate relations, which are then applied to subsequent encounters with those objects. Microanalyses of situations in which children shape their knowledge about the world reveal patterns associated with children's participation in social structures, which guide their attention and interactions with the objects themselves. In their study of five dyads, de Barbaro, Johnson & Deák [52] document the details of the transition to decentered object perception in terms of the shift in motor and social engagements from dyadic to triadic interactions. While for some researchers, an ability to engage in triadic interactions results from an unobserved process of conceptual reorganization [54], de Barbaro *et al.* claim that it is possible to track the way in which sensorimotor experiences in a social environment act as stepping stones toward the development of this ability.

First, four-month-old infants tend to focus all their modalities on a single target, for instance, looking at an object they are grasping or exploring haptically with their hands and mouth. At this stage, the caregiver is able to control the infant's attention easily by initiating eye contact and by moving objects. Subsequently, when they are approximately six months of age, children begin to use both hands to reach for objects actively and are able to divide their attention between grasping one object and looking at another. Curiously, they start to display negative affect when the mother removes the attended object as if the mother's intervention were viewed as disruptive, requiring attentional resources to be negotiated between the participants actively. Later still, when children are nine months old, fluent shifting of attention among multiple targets becomes more pronounced, and certain interactive routines, with both objects and parents, become stable. Finally, when they reach the age of 12 months, children are able to attend to objects in parents' hands and those in their own hands and to imitate their parents’ actions, perhaps manifesting a more ‘mature’ concept of the object. In this developmental trajectory, the child's agency, multimodal first-person experience in relation to actions, and social scaffolding are all considered to be crucial.

Social involvement plays an even more important role in Di Paolo's notion of participatory object perception [53]. He argues that it is difficult to produce a detached perception of objects (a way of regarding objects as external entities that have their own properties and are independent from the observer) by way of an individualistic exploration of the environment. While it is possible to discover cross-modal sensorimotor contingencies (I am looking at the ball while touching its surface) or interact with the same objects in the context of different actions (reaching for a ball versus throwing a ball), there is always an ‘I’ at the centre of these experiences—the perspective is strictly egocentric. As Di Paolo argues, to conceptualize objects as existing in the world and as perceptually available to other observers, perspective-taking skills are necessary; these skills develop in the context of social interactions.

Acquiring the notion of ‘object’ allows us to revisit relations from yet another angle, which recalls the division between the relational categories and entity categories discussed by Gentner & Kurtz [6]. Although according to the framework we advocate here, experienced relations are the foundations of all concepts, relational categories *sensu* Gentner require a particular trajectory of selection from experience. In multiple experiments, Gentner and her colleagues demonstrate empirically the ways in which relations are ‘at work’ in conceptual knowledge and the manner in which they arise through the process of comparison, analogical structure matching and re-representation. This process of selection causes certain relations to come to the fore, while others are relegated to the background. Acquired relational concepts become transferable among different contexts. In terms of ecological psychology, we can say that learning in one domain leads to the discovery of (latent) relations, which becomes a basis for the skills of selection in the context of subsequent acts of perception. As explained earlier, the very act of looking is never the same after learning such a new relation, because learning means that the cognitive system has been in-formed (structured) to perceive in a specific way.

Let us illustrate one possible route toward the formation of relational concepts by reference to an example of spatial relation. We may start with an experience of an object (which emerged in a dynamical process, as described above), for instance, a tea mug located to my right. It is constituted by a set of first-person experiences; for instance, when I turn my head to the right, I see the mug in my field of vision, or I can reach for it with my right hand. The mug may be replaced by another object, for example, a pen (Gentner's comparison stage). By backgrounding the identity of the object and selecting the experience of sensory-motor contingency, I can arrive at the concept ‘X being to my right’ (Gentner's analogical structure matching). This notion is an egocentric concept that depends on my own position in space. Henceforth, every act of perceiving an object in space is simultaneously an act of spatial orientation (Gentner's re-representation). Potentially, I can generalize this notion further using perspective-taking skills that have been developed during social interactions [53]. While interacting with a partner, I can recognize not only ‘X being to my right’ but also ‘X being to my partner's left’. This experience may give rise to the general concept of ‘X being to the right from the perspective of person Y’. This more complex notion is a fully relational concept that can be applied to any object and any observer.

Importantly, from our interaction-focused perspective, relational categories do not need to be constructed progressively based on nonrelational categories: relation and relata may coconstitute each other and be conceptualized simultaneously. Processes leading to the formation of the concepts of objects, such as mugs or pens, and the concepts of spatial relations are likely to be interwoven. The extraction of a common order relational structure and the subsequent re-representation to match this structure better by foregrounding the relation here is achieved in a single step: discovering latent commonalities results in the possibility of a changed direct perception, and so no re-representation is needed. The ability to select in accordance with a complex relation is thus acquired and present in the system, and the more stable it becomes due to its frequent use and connections with other relations, the more it is able to change the relative importance of the multiple relations that are operative at a given moment.

Relational concepts are examples of what Kazimierz Twardowski [55] calls analytical concepts. The distinguishing factor in this context is the imaginability of a concept. Analytical concepts are constructed out of percepts or other concepts by selecting some parts of the perceived whole that cannot be imagined on their own, i.e. without entities that have these properties or exist within certain relations (for example, having a colour, being long, or being a daughter). By contrast, entity categories are Twardowski's synthetic concepts, as they are characterized by ‘wholeness’. This difference in imaginability may be one reason why relational concepts are sometimes treated as more abstract than entity concepts.

Abstracting from concreteness, understood in terms of closeness to the individual lived experience, can occur along many dimensions, scaffolded by social routines and cultural artefacts. The trajectories of this reorganization vary widely, as does the degree of dependence on such scaffolds. This variance may lead to differences among concepts, which may be difficult to bridge by applying theories that rely solely on the outcomes of such processes and fail to recognize the trajectories of reorganization. We now turn to the perspective on the problem of a variety of conceptual structures that is enabled by the present framework.

### (c) Theories of categorization viewed as different routes for and dimensions of abstraction

Groupings of perceived objects and perceived patterns of their similarities can be viewed as manifestations of the particular conceptual relations that are involved in the present actions. Simultaneously, such groupings and patterns are able to forge new concepts (the circularity is intentional). Perceiving an object as a member of a particular category is an example of the imposition of selective constraints by a concept. When I observe the letter ‘a’, I primarily perceive the familiar shape of the letter I learned rather than the individual characteristics of this particular instance. I can recognize the letter instantly, but copying its exact graphical form would require effort. The habitual mode of perception is to view the letter through the lens of the learned concept, which highlights the prototypical characteristics of the letter and conceals the individual variation. Nevertheless, when presented with novel variants of the letter, I can update my notion of the category (i.e. concepts constrain percepts, while percepts shape concepts).

Selecting experiences that are related to the prototypical properties of the perceived objects is a mode of conceptualization that is connected with classic prototype theory [56]. Perceived similarity among members of the same category and dissimilarity among members of different categories contribute to the stabilization of category prototypes. Other modes of conceptualization are possible, for instance, by considering the individual object through the lens of previously established rules [57] or by formulating ‘folk’ theories that focus on causal structures rather than, e.g. visual structures [58]. These modes may require the use of (other) previously formed concepts to select more abstract relations. This process is possible since the boundary between percepts and concepts is permeable; they are both part of lived experience. Discovered relations can supplement existing relations, and sometimes one constellation of relations can overpower another possible organization. In the case of theory-building, the relations among already established concepts are used to produce a new concept. Let us assume that through repeated encounters, I was able to learn the concept of the letter ‘a’ and then another concept of bold font. It is possible to combine these two concepts in such a way as to form the concept of a ‘bold letter “a”’ even if I have never seen one. In this context, combination refers to a particular type of relation between the two concepts—the selection and conjunction of compatible relations.

Selection of experienced relations is always a process that occurs in the ‘here-and-now’, i.e. in contact with actions in the world as well as with first-person experience. Concepts are therefore always adapted to a situation, shaped by our goals and motivations. In a seminal work, Barsalou proposed a special category of such highly flexible concepts, which can be constructed on-the-fly, namely *ad hoc* concepts [7,8]. Nevertheless, we postulate that a gradation exists between *ad hoc* concepts constructed primarily based on momentary experience and concepts that make extensive use of latent relations discovered throughout the individual's history of interactions with the world, which are thus stabilized in many situational contexts. If, as we stipulated above, it is always impossible to separate from a context completely since our existence always features some goals, values and feelings as well as some degree of contact with the world, it is possible that Casasanto & Lupyan [59] are right in arguing that all concepts are *ad hoc* concepts and are more dynamic than ordinarily assumed. As Rosch [60] notes, we encounter the ‘*ad hoc* intrusion of world knowledge’ (p. 111), i.e. the ‘constant need for input of relevant real-world knowledge into all aspects of the models’ (p. 112).

We emphasize that the difference between the modes of conceptualization thus described does not so much pertain to an established category structure nor distinct cognitive processes, as proposed by concept heterogeneity hypothesis [61]. Rather, the difference concerns the kind of relations that are employed in subsequent perceptions. The same process can result in different types of concepts due to the particular trajectory of applying relations in question.

### (d) Conceptual hierarchies

In addition to the general theory of concepts and types of conceptual representations, a substantial body of research has been devoted to conceptual hierarchies. The same objects can be recognized as members of different categories and as involving different concepts; a particular bird may be a member of the bird category, the dove category (a subordinate category) or the animal category (a superordinate category). The notion of a basic-level category explicitly entails that some category levels are ‘natural’ or ‘default’. As Rosch *et al*. [62] argue, basic-level categories are the categories that are most informative under normal circumstances, meaning that they capture important distinctions in the structure of the environment (minimizing the similarities among categories) and reduce cognitive effort by allowing less important distinctions to fade to the background (maximizing the similarity within the category). Importantly, the set of object attributes that serves as the basis for categorization includes sensorimotor plans for interactions with that object. In line with our framework, the structure of the categories reflects the structure of our interactions with the world and our present engagements. When I recognize a graphical sign as the letter ‘a’ and not as just any ‘letter’ or the ‘letter “a” in the Times New Roman font’, it is because this conceptualization is most useful with respect to the activity of reading in which I am engaged. It could be stipulated that typical modes of interaction—in specific contexts—determine the default level of conceptualization (Borghi [63] called these modes canonical actions).

What remains significant with respect to basic-level categories according to our theoretical perspective is that there are different possible paths that can be taken to acquire category hierarchies. Once again, let us illustrate this claim using examples. First, consider the category of dogs. A child may encounter individual dogs during a walk in a park, notice the similarities among dogs and human practices involving them and form a general concept of a dog. The child's subsequent interactions with dogs are then filtered through the acquired concept, and they notice more subtle patterns of variation. This process leads to the formation of concepts related to various breeds of dog, which are subordinate to the basic-level concept of a dog. Subordinate concepts are formed only after the basic-level concept becomes stable and require additional percepts (supplemented by the existing concept). Now, imagine a child who explores objects in her home environment and learns the concepts of *table*, *chair*, *wardrobe*. Subsequently, through an understanding of a common function or a common verbal label, the child combines all these basic-level concepts into a superordinate concept of *furniture*. This shift can be made through a purely conceptual combination, without additional percepts. In both cases, basic-level concepts are acquired first, but the roles of these concepts and their relations to perception differ.

Note that the trajectories thus described are individual and need not be universal. In the second example, we can imagine the child of a carpenter being exposed to furniture production processes from a young age. For such a child, *furniture* may be a basic category that precedes its differentiation into tables, chairs and wardrobes. Such experience-dependent patterns of classification have been demonstrated repeatedly in the research, including famous experiments conducted by Bruner & Goodman [64], the influence of expertise on tool categorization [65] and conceptual hierarchies [66]. More recent studies concern differences in categorization in the context of bulimia [67]. Accounting for this individuality and the variety of experience requires research methods that range from qualitative analyses of first-person experience and microanalyses of the situations surrounding concept use to quantitative analyses of frequencies or situations and average performances.

## 4. Conclusion

We have highlighted an alternative conceptual organization of the field of research concerning concepts. We do not position this model in strict opposition to the traditional view; rather, we believe in the possibility of pluralism in cognitive science, such that various approaches are able to complement each other [68,69].

It is quite possible that feature-based, objective descriptions of phenomena are sufficient to explain some cognitive functioning in certain life situations, as successes in the field of artificial intelligence seem to confirm. However, other tasks and situations might require a shift in our basic assumptions or mandate that we ask novel questions, as they might impose other ‘demands on our “theories” of concepts’ ([60], p. 111). This group includes issues pertaining to concepts that are built on relations other than simple similarity, questions regarding the immediate pertinence and flexibility of conceptual structures to the situations at hand and questions concerning differences in individual and cultural experiences, which can lead to different conceptualizations and classifications (e.g. [70–72]). For these issues, a framework that bases the theory of concepts on a fundamental organism–environment relationality, which involves the capacities, goals and values of agents as well as their first-person experiences and that acknowledges the crucial role played by sociality in the formation of conceptual systems may be necessary.

A point that seems to be significant in this context is the range of problems for which it is important that the concepts of a ‘bottle’, a ‘book’, ‘gender’ or ‘COVID-19’ are not sufficiently and neatly rendered by an objective set of features but must necessarily include complex, dynamic relations, first-person experiences and knowledge concerning their trajectory and relative reliance on social interactions. This point is even more the case regarding objects in social ontology, such as ‘school’, a ‘US visa’, ‘the nature of my relationship with my nephew’, ‘ownership’ or ‘justice’, or concepts strongly rooted in first-person experience, such as ‘love’ or ‘fear’. For such concepts, it might simply not make much sense to search for descriptive sets of features, as they require contexts that include both external events and first-person experiences to be created, which must be done anew in each case. In these cases, elucidating the associated processes of creation and the functions that such concepts have in our actions would be more useful than their context-free and impassive descriptions (see, e.g. the research concerning the concept of ‘gender/sex’ by [73]). The interaction-based approach to concepts that we have begun to take by proposing the three claims discussed above moves in this direction.

A more general interaction-based framework seems to have begun to coalesce as an alternative to the information-processing perspective on cognitive systems in their environments [74–76]. Despite their differences, action-first or interaction-based theories of cognition, such as ecological psychology, enactivism and interactivism collaborate, more or less intentionally, in the process of developing a novel approach, building on the foundation of the organism–world relation, process ontology, emergence and the forward-looking, anticipatory character of cognition, while converging on dynamical systems theory and methods as a common ground [14,16,17,77–79]).

However, such a profound reorganization might not be necessary for certain problems. In these cases, it would be sufficient to show that the relational processes of selection that occur in the context of situated actions and within the social realm can offer explanations that are more useful for some types of phenomena. In the sections above, we tried to show the links between this approach to research and traditional methodologies and to indicate how our ability to understand some phenomena can be facilitated by the interactive framework. Equally interesting would be the cases that are difficult to render according to the fully dynamical, interaction-based approach, thereby suggesting the complementarity of frameworks. It might well be the case that the dependence of some conceptual areas on replicable forms such as language and formal systems makes them naturally more amenable to the kinds of explanations that are more closely related to information-processing approaches, and that this form of explanations would indeed be sufficient for some purposes.

This situation is—possibly—the case for some of the language-to-language tasks, for which it seems sufficient to rely on statistical co-occurrences of patterns, omitting the connection to interactive dynamical processes or first-person experiences. For the sake of brevity, we refrained from discussing the role of language in establishing novel conceptual relations, and making them public (but see [30,80–88]). However, it should be clear that patterns of word co-occurrences will always be the surface manifestations of dynamical process on many timescales and levels, and it seems important that we are aware of that fact if we are to be able, when necessary, to retrace these threads to social interaction and experience. The most recent examples of the large-scale language models performing various tasks—such as translation, content classification, linking and content summarizing—testify to the power of relying on such statistical patterns on the one hand, but on the other to the importance of being vigilant regarding the biases that such an approach may introduce [89,90], which, in turn, may impinge on real-life human interactions. Being able to find a path back to basic experiences in interaction is crucial; if we fail to do so, ‘does not all “significance” depart from the situation?’ ([45], p. 19).

The approach explicated here may allow cognitive science to become more sensitive to the idiosyncrasy of human experience. The three tenets discussed make more evident the way in which, within culturally sanctioned habits (as stabilized by conventionalized linguistic expressions or routines), each individual may embody a different pattern of relations/dependencies, which might be prone to different organizations. Focusing on one pattern or another may testify to its frequency, usefulness and systemic coherence; however, it also testifies to the power and privilege inherent in the ability to access the means of making some concepts and patterns dominant, thereby appropriating certain conceptual orders. Culturally established lines may thus be imposed on and overpower felt relations. Such a realization that modes of organization are temporary selections among the alternative latent organizations of experience, which have certain functions in specific life forms and language games, may make it possible to acknowledge the other such modes and develop a better mutual understanding. Constant vigilance regarding alternative organizations of the field of experiences in various contexts opens us up to others as sources of knowledge regarding those organizations as well as to communication in the form of seeking points of compatibility and complementarity rather than searching for a total alignment on the ultimately ‘correct’ conceptual organization.

## Data Availability

This article has no additional data.

## Footnotes

1 See, e.g. the recent Frontiers Research Topic edited by di Paolo, Heras-Escribano, Chemero & McGann [12].

2 This intrinsic nature can be understood as a result of the biological preparedness of the ‘field’ constituted by the species–environment relations (which can be shaped further by an individual organism's experience) or as a potential differentiation that is inherent in physical reality for *any* potential living organism. On our reading of James, these two senses are not differentiated.

3 By the environment engaging an agent, we refer both to the habitat—the evolutionary important ‘sum’ of possible experiences to which species adapt—and to the Umwelt—the surroundings of a particular individual. This useful distinction was made in the context of ecological psychology by Baggs and Chemero [31, p. 2175] and it ‘allows us to understand the environment in realist terms while acknowledging that individual living organisms are phenomenal beings’.
